# Notes on the ecology of rolled-leaf hispines (Chrysomelidae, Cassidinae) at La Gamba (Costa Rica)^1^

**DOI:** 10.3897/zookeys.332.5215

**Published:** 2013-09-19

**Authors:** Michael Schmitt, Meike Frank

**Affiliations:** 1 Ernst-Moritz-Arndt-Universitaet, Allgemeine & Systematische Zoologie, Anklamer Str. 20, D-17489 Greifswald, Germany; 2 Blumenthalstr. 68, D-50668 Köln, Germany

**Keywords:** Insecta, Coleoptera, Chrysomelidae, *Cephaloleia*, *Chelobasis*, Zingiberales, Costaceae, Heliconiaceae, Maranthaceae, Musaceae, Zingiberaceae, synecology, host plant, Costa Rica

## Abstract

A total of 301 adult hispine beetles of the genera *Cephaloleia* and *Chelobasis* were found in rolled leaves of plants of 17 species of Zingiberales (families Costaceae, Heliconiaceae, Maranthaceae, Musaceae, and Zingiberaceae) during a field study at La Gamba, Golfito region, Costa Rica. Of these beetles, *Cephaloleia belti* was recorded from 12 potential host plant species, *C. distincta from* 7, *C. dilaticollis* from 5, *C.*, *Chelobasis bicolor*, *C. championi*, and *C. histrionica* from 3, *Chelobasis perplexa* and *C. instabilis* from 2, whereas *C. trivittata* from only one. Of the plant species, *Heliconia latispatha* had 7 beetle species in its leaf rolls, *Calathea lutea* had 5, *H. imbricata* and *H. rostrata* had 4, *H. stricta* and *Musa paradisiaca* had 3, *H. wagneriana* had 2, while on *H. vaginalis*, *H. danielsiana*, *H. densiflora*, *H. longiflora*, *Calathea crotalifera*, *C. platystachya*, *Goeppertia lasiophylla*, *Alpinia purpurata*, *Costus pulverulentus* and *Costus barbatus*, *H. densiflora*, *H. vaginalis*, and *H. danielsana* only hispines of one species were found.

*Cephaloleia belti* occurred together with beetles of six other hispine species, whereas *Cephaloleia trivittata* never shared a leaf roll with another hispine species. The remaining beetle species aggregated with one to four other hispines. Adults of *C. belti* and *C. championi* were frequently seen, occasionally also with *C. dilaticollis*, *C. histrionica*, *and Chelobasis perplexa*, to co-occur with the carabid *Calophaena ligata* in the same leaf roll without any sign of interspecific aggression.

A comparison of host choices and the phylogeny of the hispines and of their host plants revealed no signs that beetles used species level phylogenetic relationships within the Zingiberales to select food plants. Obviously, within this plant order, rolled-leaf hispines choose their plant hosts in a nearly opportunistic manner. Seemingly, they use differences among plants at higher taxonomic levels but within the Zingiberales, the availability of young – rolled – leaves might be the actual decisive factor.

## Introduction

Since the nineteenth century it has been known to science that beetles of a (probably monophyletic: McKenna and Farrell 2005) subclade of the traditional Hispinae (hispine Cassidinae) develop as larvae and live as adults inside the tubes formed by rolled leaves of Zingiberales plants (Baly 1885: 8; Maulik 1919: 12). As larvae and adults of these beetles produce characteristic feeding tracks, Wilf et al. (2000) inferred from similar tracks on fossil Zingiberales leaves that this special type of plant-herbivore interaction evolved as early as the late Cretaceous, about 66 Mio years ago. However, García-Robledo and Staines (2008) raised doubt and discussed an origin of this behaviour ca. 20 Mio years later because other insects were found producing similar feeding tracks, e.g. Lepidoptera larvae in the families Pyralidae and Choreutidae and weevils (Coleoptera: Curculionidae) of the genus *Anopsilus* Kirsch, 1869. The use of rolled leaves as habitat by leaf beetles is in the New World restricted to species of the tribes Arescini and Cephaloleiini, whereas in Indonesia also a *Hispodonta* sp. has been observed in rolled leaves of *Musa* and *Zingiber* (see Staines 2004). A remarkable body of publications treat the development, ecology, phylogeny, and taxonomy of these beetles (see Staines 2004; Chaboo 2007 for an extensive literature review). According to Seifert (1982), “the insect fauna associated with *Heliconia* plants is one of the most intensively studied of all (non-cultivated) Neotropical insect-plant associations”. Strong (1983) described concisely the biology of the rolled-leaf hispines, using *Chelobasis bicolor* as the main example. A more detailed description of the natural history of *Cephaloleia*-species, based on field observations and laboratory investigations, is given by García-Robledo et al. (2010). These beetles spend most of their lives inside the rolled leaves of Zingiberales plants, on which they feed. The larvae are flattened and move and feed between the layers of the rolled leaves. Pupation takes place at various places on the host plants. Also the adults of these beetles can move between the layers of the leaf rolls, in many species they are flattened, and all are spineless, in contrast to the majority of hispines that usually live on the surface of leaves as adults.

Our primary aim was to assess the number of rolled-leaf hispines species and their abundances in the area of the biological field station “La Gamba” in the Golfito region of Costa Rica and to compare our findings with those from the “La Selva” biological station (Strong 1977a, b, 1982a, b; García-Robledo et al. 2010; Staines 2011; García-Robledo et al. 2013) and from sites in lowland central Panama (Descampe et al. 2008, Meskens et al. 2008). In addition, we collected data on putative host preferences and inter-specific aggregations of these beetles, including those with the carabid *Calophaena ligata*.

## Study site

All field work was performed at the La Gamba biological station, Costa Rica (Puntarenas), 8 km NNW of the city of Golfito, 8°42'61"N, 83°12'97"W, 70 m a.s.l., and ca. 8 km off the coast of the Golfo Dulce. The station is located at the edge of the Piedras Blancas National Park and is run by the Verein zur Förderung der Tropenstation La Gamba (society for the furtherance of the La Gamba tropical field station), based at the University of Vienna (Austria) (www.lagamba.at). Numerous plants of the order Zingiberales grow in the 2200 m^2^-garden of the station. Most individual plants are accurately identified to species and labelled. Botanists from the department of Tropical Ecology and Animal Biodiversity at the University of Vienna are responsible for the scientific supervision of the station, and the accurate identification of the plants in the garden. The station is situated between secondary and primary forest areas to the west, south, and east, and adjoins agriculturally managed areas, mostly pastures and oil palm plantations, to the north. Several trails through the forest allow access to sites inside the forest, e.g. to clearings where the host plants in this study were most abundant.

## Methods

As no other plants at La Gamba formed rolled young leaves, we censused only Zingiberales plants in the station’s park and along trails for rolled leaves at 15 day intervals within the months of January through April, 2009. We unrolled 120 rolled leaves and recorded the macrofauna found in them. The hispine leaf beetles and other arthropods were collected from the leaves and taken to the station. We also kept records of findings of hispine larvae, eggs, and feeding tracks. In some cases we took photographs as exemplars. Some of the hispines were killed and mounted for identification, others were stored in ethanol. The whole material is still with the senior author for further examination of the non-hispine species. It will be deposited at the Museo Zoológico of the Universidad de Costa Rica at San Pedro, voucher specimens will be deposited at Zoologisches Forschungsmuseum Alexander Koenig, Bonn (Germany).

We identified the beetles by comparison with identified specimens in the collection at the Instituto Nacional de Biodiversidad (INBio) at Santo Domingo de Heredia and by means of published keys and original descriptions (Baly 1885; Staines 1996, 2009). We identified host plants using the labels in the station’s park or by using the keys in Weber et al. (2001). With regard to plant taxonomy and nomenclature, we followed Borchsenius et al. (2012), GRIN (2013), and Tropicos (2013). For the statistical analysis we used the Statistical Package for Social Sciences (SPSS).

## Results

**Figure 1.**
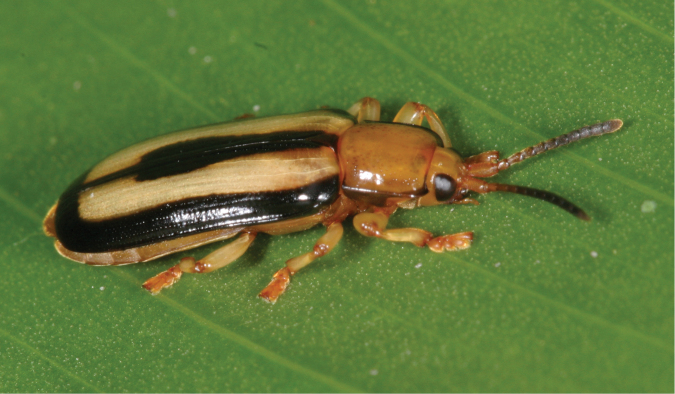
*Cephaloleia championi* from an unrolled *Heliconia*-leaf at La Gamba. M.Schmitt phot.

**Figure 2.**
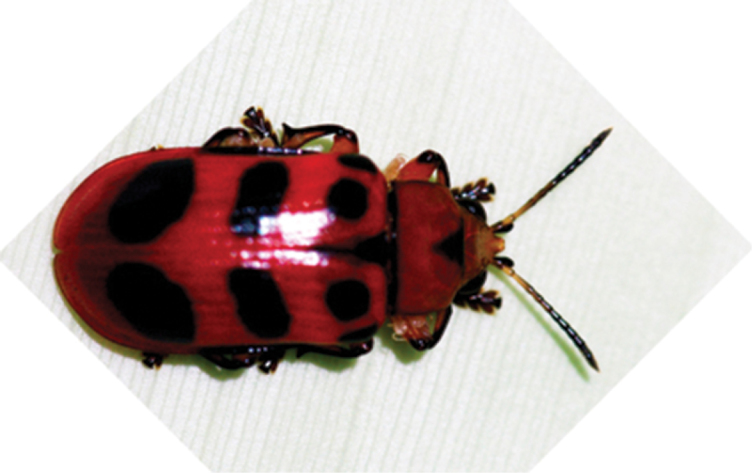
*Chelobasis bicolor*, La Gamba. M. Frank phot.

We found 301 individuals of nine species of hispines, all from two genera, *Cephaloleia* Chevrolat, 1837 and *Chelobasis* Grey, 1832. These were the Cephaloleiini *Cephaloleia belti* Baly, 1885, *Cephaloleia championi* Baly, 1885 (Fig. 1), *Cephaloleia dilaticollis* Baly, 1858, *Cephaloleia distincta* Baly, 1885, *Cephaloleia histrionica* Baly, 1885, *Cephaloleia instabilis* Baly, 1885, *Cephaloleia trivittata* Baly, 1885 and the Arescini *Chelobasis bicolor* Gray, 1832 (Fig. 2) and *Chelobasis perplexa* Baly, 1858. They were collected from 17 identified and at least two unidentified Zingiberales species: *Alpinia purpurata* (Zingiberaceae), *Calathea crotalifera*, *Calathea lutea*, *Calathea platystachya*, and *Goeppertia lasiophylla* (Marantaceae), *Costus barbatus* and *Costus pulverulentus* (Costaceae), *Heliconia danielsiana*, *Heliconia densiflora*, *Heliconia imbricata*, *Heliconia latispatha*, *Heliconia longiflora*, *Heliconia rostrata*, *Heliconia stricta*, *Heliconia vaginalis*, and *Heliconia wagneriana* (Heliconiaceae), and *Musa paradisiaca* (Musaceae). The numbers of the collected beetles and the respective potential host plants are given in Table 1. The beetle records are unequally distributed over their potential host plants. Of all species found in more than 20 individuals and on more than one potential host plant, a marked majority of records are from one or few of their potential host plants.

**Table 1.** Numbers of collected hispines and their potential host plants at La Gamba<br/>

	***Cephaloleia belti***	***Cephaloleia championi***	***Cephaloleia dilaticollis***	***Cephaloleia distincta***	***Cephaloleia histrionica***	***Cephaloleia instabilis***	***Cephaloleia trivittata***	***Chelobasis bicolor***	***Chelobasis perplexa***
*Alpinia purpurata* (Zingiberaceae)				**14**					
*Calathea crotalifera* (Marantaceae)			**8**						
*Calathea lutea* (Marantaceae)	**2**	**33**	**15**		**6**				**1**
*Calathea platystachya* (Marantaceae)		**3**							
*Costus barbatus* (Costaceae)	**1**								
*Costus pulverulentus* (Costaceae)							**3**		
*Goeppertia lasiophylla* (Heliconiaceae)	**1**								
*Heliconia danielsiana* (Heliconiaceae)	**1**								
*Heliconia densiflora* (Heliconiaceae)	**2**								
*Heliconia imbricata* (Heliconiaceae)		**1**		**1**	**6**			**1**	
*Heliconia latispatha* (Heliconiaceae)	**57**		**3**	**3**	**4**	**3**		**4**	**3**
*Heliconia longiflora* (Heliconiaceae)	**4**								
*Heliconia rostrata* (Heliconiaceae)	**36**		**4**	**1**		**1**			
*Heliconia stricta* (Heliconiaceae)	**12**			**1**				**1**	
*Heliconia vaginalis* (Heliconiaceae)	**2**								
*Heliconia wagneriana* (Heliconiaceae)	**41**			**3**					
*Musa paradisiaca* (Musaceae)	**2**		**1**	**1**					
Zingiberales indet.	**9**			**1**			**5**		
Totals	**170**	**37**	**31**	**25**	**16**	**4**	**8**	**6**	**4**

The unidentified Zingiberales grew outside the station garden and lacked inflorescences. We could not identify them using Weber et al.’s (2001) key.

**Figure 3.**
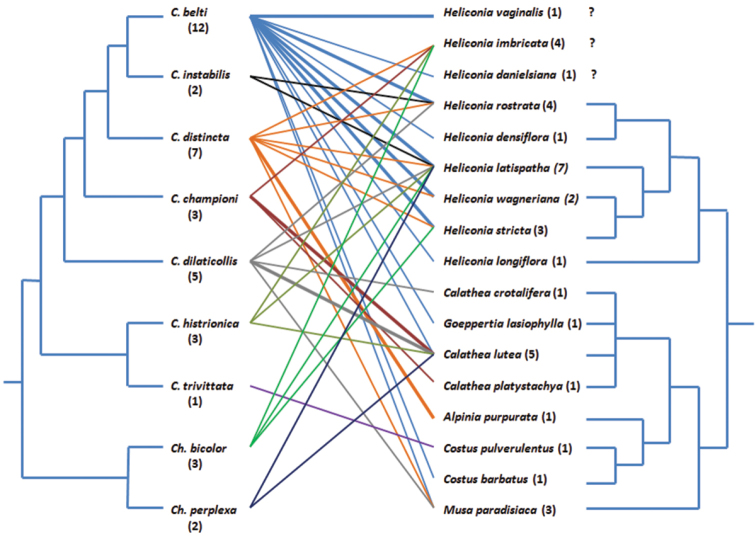
Food web of the rolled-leaf hispines of La Gamba and their possible food plants based on the data in Table 1, drawn by hand using MS Powerpoint. Bold lines indicate more than ten beetle records on the respective plant. Numbers in parentheses give the number of plant or beetle “partners”, respectively. Beetle cladogram after McKenna and Farrell (2005), plant cladogram combined after Marouelli et al. (2010) and Janssen and Bremer (2004). *Heliconia vaginalis*, *Heliconia imbricata*, and *Heliconia danielsiana* were – for some unknown reason - not included in these phylogenetic analyses.

We opposed a molecular cladogram of the genus *Cephaloleia* (from McKenna and Farrell 2005) with that of their potential host plants (combined from Marouelli et al. 2010 and Janssen and Bremer 2004) to reveal possible matches between beetle and plant phylogeny and visualise the insect-plant relations as a food-web (Fig. 3). There is no obvious preference of hispines for closely related plants, nor is there an apparent correlation of the phylogenetic relationship of the beetles and that of the plants on which we found them. It becomes clear, however, that the rolled-leaf hispines had preferences for certain Zingiberales species even if they were found on a much broader spectrum of possible food plants.

Rolled-leaf hispines, with the exception of *Cephaloleia trivittata*, co-occurred at La Gamba with at least one other species in the same leaf roll, *Cephaloleia histrionica* with only one other species. *Cephaloleia belti* was found in the same leaf roll together with *Cephaloleia championi*, *Cephaloleia dilaticollis*, *Cephaloleia distincta*, *Cephaloleia instabilis*, *Chelobasis bicolor* and *Chelobasis perplexa*. The remaining hispines shared leaf rolls with two to four other hispine species. The numbers differed considerably, as shown in Table 2. Statistical tests – we used Chi^2^ - could only be performed for the four most abundant species, as of the remaining species we found too few individuals.

**Table 2.** Co-occurrence of rolled-leaf hispines in the same leaf roll at La Gamba.<br/>

	**Found in the same leaf roll together with:**										
	***Cephaloleia belti***	***Cephaloleia championi***	***Cephaloleia dilaticollis***	***Cephaloleia distincta***	***Cephaloleia histrionica***	***Cephaloleia instabilis***	***Cephaloleia trivittata***	***Chelobasis bicolor***	***Chelobasis perplexa***	**a**	**b**
*Cephaloleia belti* (n = 170)	**120**	**1**	**17**	**26**		**10**		**15**	**8**	Chi^2^: 286.7 p: <0.001	Chi^2^: 9.878 p: 0.003
*Cephaloleia championi* (n = 37)	**6**	**17**	**12**		**8**				**1**	Chi^2^: 6.581 p: 0.087	Chi^2^: 1.884 p: 0.227
*Cephaloleia dilaticollis* (n = 31)	**15**	**11**	**14**	**1**					**1**	Chi^2^: 0.650 p: 0.778	Chi^2^: 3.600 p: 0.080
*Cephaloleia distincta* (n = 25)	**11**		**1**	**15**		**2**		**2**	**1**	Chi^2^: 17.20 p: 0.001	Chi^2^: 0.000 p: 1.000
*Cephaloleia histrionica* (n = 16)		**4**			**12**					Chi^2^: n.a. p: n.a.	Chi^2^: n.a. p: n.a.
*Cephaloleia instabilis* (n = 4)	**4**			**3**						Chi^2^: n.a. p: n.a.	Chi^2^: n.a. p: n.a.
*Cephaloleia trivittata* (n = 8)							**8**			Chi^2^: n.a. p: n.a.	Chi^2^: n.a. p: n.a.
*Chelobasis bicolor* (n = 6)	**4**			**3**				**1**		Chi^2^: n.a. p: n.a.	Chi^2^: n.a. p: n.a.
*Chelobasis perplexa* (n = 4)	**3**	**1**	**1**	**2**						Chi^2^: n.a. p: n.a.	Chi^2^: n.a. p: n.a.

Numbers in the cells indicate the numbers of beetles given in the first column co-occurring with beetles of species given in the same horizontal row, e.g.: 26 of the 170 *Cephaloleia belti* were found together with *Cephaloleia distincta*. As in several cases beetles of more than two species were found in one leaf roll, the checksums in these cases are higher than the total numbers given in the first column. White cells mark the exclusively conspecific aggregations, pink cells indicate observed records, yellow and blue cells mean that these theoretically possible co-occurrences have not been found in the present study. Column **a**: Chi^2^ and p-value for an aggregation of the species in the line with conspecifics or with any other species. Column **b**: Chi^2^ and p-value for an aggregation of the species in the line with the pooled other hispines.

**Figure 4.**
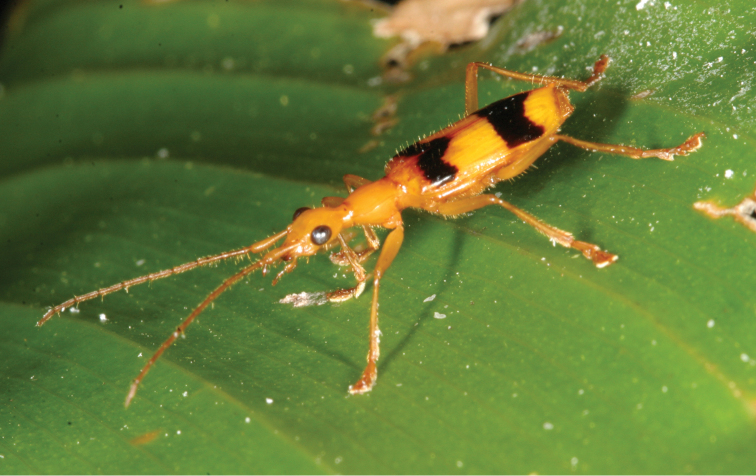
*Calophaena ligata* on an uncoiled *Calathea lutea*-leaf. M. Schmitt phot.

Individuals of the ground beetle, *Calophaena ligata* Bates, 1883 (Carabidae: Harpalinae, Fig. 4) (29 individuals) were found on *Calathea lutea* exclusively, co-occurring with *Cephaloleia belti* (11), *Cephaloleia championi* (19), *Cephaloleia dilaticollis* (7), *Cephaloleia histrionica* (4), and *Chelobasis perplexa* (1). Four *Calophaena ligata*-individuals were found in a single leaf roll without any hispine company. These beetles always sat on the inner surface of the leaf roll and were never found between two layers of a roll. We could never observe them feeding inside the leaf roll, nor could we find them on an uncoiled Zingiberales leaf at daylight.

## Discussion

Our small set of observations show that in the use of host plants there are generalists and specialists among the hispine beetles found in the rolled leaves of Zingiberales at La Gamba. This is in general concordance with earlier investigations (Strong 1977b; Staines 2011; García-Robledo and Horvitz 2012a, b). Our sample is by orders of magnitude smaller than that of Strong (let aside that of García-Robledo et al. 2013), so that comparisons must be judged cautiously. At La Gamba, it seems that in species where we found more than 20 individuals on more than one plant species, the beetles clearly preferred some plants over others, as indicated by their numbers in the sample. We found *Cephaloleia belti* on 12 Zingiberales plants, but only on five *Heliconia*-species (*Heliconia latispatha*, *Heliconia rostrata*, *Heliconia stricta*, *Heliconia vaginalis*, *Heliconia wagneriana*) were there more than 10 individuals, on the remaining seven plants there were only one or two. Similarly, 37 individuals of *Cephaloleia championi* occurred on three plant species, but on *Calathea lutea* there were 33, whereas on *Calathea platystachya* and *Heliconia imbricata* we found only three and one individual(s), respectively. Other examples are *Cephaloleia dilaticollis* and *Cephaloleia distincta*, see Tab. 1. However, due to the many empty cells in Table 1, only for *Cephaloleia belti* could a statistical test be performed. It revealed a Chi^2^ of 524.84 and a p < 0.001 for the distribution of the beetles over the plant species being caused by chance. Since the findings with only four or fewer beetle individuals cannot conclusively indicate that the beetles used the places where we found them as feeding sites, we address these plants as “potential host plants”. We regularly found feeding traces on the surface of the uncoiled leaves. Some traces could be assigned to certain hispine species, following Strong (1977b). However, not all feeding traces were clearly species-specific, and often we found beetles not actually feeding, and in assemblages of several species. Nevertheless, it seems that the rolled-leaf hispines have the ability to exploit plants of more than one family, in contrast to Strong’s (1977b) suggestion. This is corroborated also by Descampe et al. (2008) who found in Panama that the 8 rolled-leaf hispines in their study attacked 4 to 9 of the 11 species of Heliconiaceae and Marantaceae investigated. Also, Meskens et al. (2008) conclude that the host plant spectrum of the rolled-leaf hispine species is broader than previously thought. García-Robledo and Horvitz’s (2012a, b) found in a choice experiment that *Cephaloleia dilaticollis* – classified as a generalist – accepted *Alpinia purpurata*-leaves as oviposition sites to the same degree as their native host plant *Renealmia alpinia* (Zingiberaceae). At la Gamba, we found no individual of this species in *Alpinia* leaf rolls. This could mean that in the field interspecific competition could have prevented *Cephaloleia dilaticollis* from entering the *Alpinia* leaf rolls.

It is evident that of all Zingiberales species at La Gamba, *Heliconia latispatha* harboured the greatest number of rolled-leaf hispine species (7), followed by *Calathea lutea* (5) and *Heliconia imbricata* and *Heliconia rostrata* (4 each). The species richness of *Heliconia latispatha* is well documented (Strong 1977a). However, the markedly highest number of hispines on these *Heliconia*-species were *Cephaloleia belti*. Only on *Calathea lutea* we found two hispine species (*Cephaloleia championi* and *Cephaloleia dilaticollis*) in roughly comparable numbers.

Due to limitations in sample size, the outstanding case of *Cephaloleia trivittata* on *Costus pulverulentus* does not convincingly demonstrate a high degree of specialisation, especially since we found five *Cephaloleia trivittata* individuals on unidentified Zingiberaceae. We can interpret this finding at best as an indication of a possible specialist among the species investigated.

Another remarkable observation is that *Cephaloleia distincta* obviously prefers *Alpinia purpurata* but uses sporadically up to six other Zingiberales species. This could mean that *Cephaloleia distincta* has a high potential to shift host plants when necessary. Differing from Staines (2011), we did not find *Cephaloleia distincta* on *Calathea* species, while so far this hispine was not reported from *Alpinia*. *Alpinia purpurata* is introduced to the Neotropics, it is native in Malaysia, New Guinea and possibly other parts of South East Asia (GRIN 2013). García-Robledo and Horvitz (2012a, b) did not use *Cephaloleia distincta* in their experiments. Nevertheless, as these authors have found *Cephaloleia distincta* exclusively on *Heliconia mariae*, this beetle is certainly not a generalist, even if we have found single individuals on other Zingiberales spp (see Table 1). Further and more detailed studies are necessary to decide whether our finding was caused by chance (e.g. by factors occurring only in the season in 2009 when we collected beetles), or if they indicate a special case of shift to a novel host plant by a rolled-leaf hispine.

As Fig. 3 shows, there is seemingly no phylogenetic pattern in the hispine-Zingiberales relations at La Gamba. Even if we consider only those relations based on more than ten beetle records per plant species, indicated by the bold lines in the figure, phylogenetic relatedness of the plants or the beetles involved does not seem to play any role. Otherwise, we would have found that closely related beetles use closely related plants. It may well be that phylogenetic factors determine food plant choice on a broader scale so that a phylogenetic pattern, as McKenna and Farrell (2005) found, becomes obvious only on a more inclusive taxonomic level. Alternatively, these factors affect other aspects of feeding behaviour such as choice of the type of plant tissue exploited or the site on the plant where the beetle or its larva actually feeds. As rolled leaves are a limited resource (Seifert 1982; Strong 1977b - even in an area where Zingiberales are planted, as in the station’s garden), their actual availability does certainly influence the beetles’ choice of host plants. García-Robledo and Horvitz (2009) found that *Cephaloleia*-individuals of four species (*Calathea dorsalis*, *Calathea erichsonii*, *Calathea fenestrata*, *Calathea placida*) reacted positively to scents of Zingiberales leaves and discriminated in most of the experiments between different plant species (e.g., their host plant against a non-host plant). In the light of these findings, our results could indicate that the chemical signals of possible host plants do not reflect their phylogenetic relationships. Wink (2003) found several such cases in his investigation on secondary metabolites of Fabaceae, Solanacceae, and Lamiaceae.

We could observe feeding only occasionally. Therefore, it is by no means certain that the associations we report here represent indeed trophic interactions. García-Robledo et al. (2013) studied plant-herbivore networks between rolled leaf hispines and Zingiberales over two years at La Selva. They identified the plants digested by the beetles using a three-locus DNA barcode. Our selection of plant species and the beetle species we recorded do not match exactly those of García-Robledo et al. (2013). Nevertheless, our results concur fairly well with theirs: *Cephaloleia belti* and *Cephaloleia dilaticollis* appear as generalists feeding on plants of more than one Zingiberales family with a preference for Heliconiaceae and Marantaceae, respectively (Table 1, see also García-Robledo and Horvitz 2012a), *Chelobasis bicolor* is restricted to Heliconiaceae. We found *Cephaloleia trivittata* on *Costus pulverulentus*, a plant these beetles had not consumed in García-Robledo et al.’s (2013) study. Given the low number of three individuals in our sample, this difference is probably biologically insignificant. It is certainly more important that also the plant-herbivore network presented by García-Robledo et al. (2013) does not show a clear phylogenetic pattern when we applied the beetle relationships as given in McKenna and Farrell (2005) and the plant relationships as presented in Marouelli et al. (2010) and Janssen and Bremer (2004).

Strong (1977b) found a correlation between the number of hispine species exploiting plants of the different Zingiberales families and the number of plant species within these families. We compared his finding with our results and found a good correspondence. It might be of interest that at La Gamba we found the same number of beetles species (8) as Strong found at La Selva on more *Heliconia*-species (9 instead of 5). This difference could indicate that the hispine diversity at La Gamba is lower than could be expected. Strong (1977a) found in his study of species richness of *Heliconia latispatha* herbivores the ratio of actual and possible feeders among the rolled-leaf hispines is 4:5 at Palmar Sur and 4:6 at Golfito, two study sites in the vicinity of La Gamba. This relation is 7:9 at La Gamba, which lies exactly between the two other values. Nevertheless, we should keep in mind that of the 77 individuals found on *Heliconia latispatha*, 57 belonged to *Cephaloleia belti*. Descampe et al. (2008) report a similar relation from Panama. They found – among others – 289 individuals of *Cephaloleia belti* and 44 of *Cephaloleia instabilis* on *Heliconia latispatha*, but none of *Cephaloleia dilaticollis*. At La Gamba, we had three individuals of the two latter species each on *Heliconia latispatha*.

The other possible meaningful result is that we found four adult beetles of three species in banana leaf rolls. Banana (*Musa x paradisiaca*) was introduced to this area of Central America by man ca. 120 years ago (Vandermeer 1983), *Alpinia* certainly not earlier, as it is used as an ornamental plant by people who worked on banana plantations. The land immediately to the north of the La Gamba station was a banana plantation until the 1980s, when United Brands abandoned the region (Hein 1990: 228). Our observations – albeit minute – could point to a beginning integration of *Musa* spp. into the food web of hispines and Zingiberales. Staines (1996) recorded two *Cephaloleia* species on banana, García-Robledo and Horvitz (2012a, b) found that *Cephaloleia belti* even preferred feeding on *Musa velutina* over their native host plants in experiments. Nevertheless, in these experiments *Cephaloleia belti* did not lay eggs on *Musa*-leaves. Since we found only four adults on *Musa*, we do not draw any further conclusion. It would be worth checking banana plants on cultivated areas near the station La Gamba for utilisation by hispines.

Strong (1982a, b) reported that several species of rolled-leaf hispines co-existed harmoniously on the same food plant and even in the same leaf roll. We, too, often found individuals of more than one hispine species inside the same leaf roll. However, it is remarkable that different hispine species tended to co-occur inside the leaf rolls considerably less often than expected by chance (see Table 2). *Cephaloleia belti* showed euryoecious behaviour not only with respect to food plants but also to tolerated allospecifics. We found individuals of this species in assemblages together with six other rolled-leaf hispines, but never with *Cephaloleia histrionica* or *Cephaloleia trivittata*. Moreover, 120 (71 %) of the 170 individuals discovered, had no other hispine companion in “their” leaf roll (which could be expected by chance only with a probability of less than 0.001). Similarly, 12 (75 %) of the 16 *Cephaloleia histrionica* lived in leaf rolls as the only hispine. Of course, the low numbers of, e.g. *Chelobasis bicolor* and *Chelobasis perplexa*, and *Cephaloleia instabilis* allow only tentative conclusions. *Cephaloleia championi*, *Cephaloleia distincta*, and *Chelobasis perplexa* showed a medium level of interspecific tolerance, *Cephaloleia dilaticollis*, *Cephaloleia instabilis*, *Chelobasis bicolor* and *Cephaloleia histrionica* a decreasing lower level, whereas *Cephaloleia trivittata* could be a rare example not only of monophagy (on *Costus pulverulentus*) but also of interspecific intolerance. That the probability of error for *Cephaloleia distincta* to be found together with another hispine species is 0.001 is no reliable evidence for a marked interspecific intolerance since 11 individuals co-occurred with *Cephaloleia belti* (so that the p-value for an aggregation of *Cephaloleia distincta* with any other hispine species is 1.000).

Although it was already reported by Baly in 1885 that *Cephaloleia*-individuals were found “often in company with species of *Calophaena* (Carabidae)” (p. 8), it was to our knowledge not mentioned in the modern papers on the biology of rolled-leaf hispines. We found 29 individuals of *Calophaena ligata* in the lumen of *Calathea-lutea*-leaf-rolls. Obviously, they co-existed harmoniously with the hispines in their leaf rolls (*Cephaloleia belti*, *Cephaloleia championi*, *Cephaloleia dilaticollis*, *Cephaloleia histrionica*, and *Chelobasis perplexa*). The genus *Calophaena* belongs to the tribe Harpalini, which comprises many phytophagous species (see, e.g., Lawrence and Britton 1994: 87). One could, therefore, presume that adults of *Calophaena ligata* feed on the plants rather than on hispine beetles. Possibly, they could occasionally prey upon larvae of rolled-leaf hispines. We cannot exclude this possibility, but we regard it as very unlikely because we never saw hispine larvae exposed on the inner surface of a leaf roll (but always feeding between two layers of a rolled-leaf), whereas we never detected one of the carabid beetles in the narrow space between two leaf layers. The adults of *Calophaena ligata* are more than twice as long as, e.g., *Cephaloleia championi* adults, and their body appendages are long and slender (Fig. 4), in contrast to the stout legs and antennae of the hispines. Although the body of the *Calophaena* beetles is depressed, as compared to epigeal ground beetles, it seems that they would have problems if they intended to hunt between leaf layers of Zingiberales plants. We speculate that the co-occurrence of *Calophaena ligata* and certain rolled-leaf hispines is a result of parallel host plant choice rather than of interspecific beetle attraction or exclusive interspecific tolerance. The syn-ecological relation of rolled-leaf hispines and *Calophaena* ground beetles remains enigmatic. We suspect that there is hardly any direct interaction but that the individuals of these two beetle families meet accidentally.

After all, it is interesting to note that *Calophaena*-individuals have exclusively been found on *Calathea lutea* plants. Daniel Blanke reported in his unpublished diploma thesis (“Autökologie der Laufkäfer der Gattung *Calophaena* – Coleoptera, Carabidae – im Piedras Blancas Nationalpark, Costa Rica”, University of Bonn 2010, supervised by M.S.) that he had found 389 individuals of this species at La Gamba, of which 387 were discovered on *Calathea lutea*. He saw them moving around on lower leaf surfaces and gnawing at the base of leaves at dark. He speculates that the ground beetles take up flavonoids from the plant and use them to produce their aposematic colouration (see Fig. 4). This idea appears plausible since *Calathea lutea*-leaves are outstandingly rich among Zingiberales in flavonoid content (Williams and Harborne 1977). Possibly, also *Cephaloleia championi*, which shows a similar colour pattern as *Calophaena ligata* (see Fig. 1), prefers *Calathea lutea* over other Zingiberales due to the high content in flavonoids.

The core conclusions from our results are: The rolled-leaf hispines at La Gamba have been found on Zingiberales, as already known from other regions in Central America (La Selva, Panama). However, we did not systematically check other plants. Among the beetles we collected were some with a broader spectrum of potential host plants, above all *Cephaloleia belti*, while other species live on fewer plant species or even only on one (*Cephaloleia trivittata* on *Costus pulverulentus*). However, the many observations of few individuals or singletons of a beetle species on several Zingiberales when the majority of their conspecifics was found on one or only a few other Zingiberales underpin the statement of Descampe et al. (2008) that the host plant spectrum of the rolled-leaf hispines is certainly broader than assumed in Strong’s earlier publications. Similar conclusions apply to the multi-species assemblages, which we often discovered. However, the high proportion of individuals found without allospecific company, even if the minority obviously is interspecifically tolerant, could mean that the rolled-leaf hispines prefer un-occupied leaf rolls over occupied ones.
